# The Institutional Worker

**Published:** 1918-09-14

**Authors:** 


THE
### INSTITUTIONAL WORKER
**Being a Special Supplement to "The Hospital"**

### OUR BUREAU OF INFORMATION.

### Rules for Correspondents.

1. Every letter must be accompanied by the coupon to be cut from the back cover (inside page) of The Hospital, current issue, and must contain the name and address of the correspondent with pseudonym for publication if desired. Replies by post cannot be given save under exceptional circumstances at the Editor's discretion.

2. Letters from Approved Homes in reply to special needs published in the Bureau should state terms and full particulars, and be sent prepaid under cover to the Editor of the Bureau with name written across coupon for identification.

3. Proprietors of Homes which have not yet been entered on the List of Approved Homes, but have spare accommodation likely to suit special needs, are invited to write for an application form for registration. The fee for registration, which includes two announcements of the Home in the Bureau and other privileges, is 10s.

4. All communications to be addressed to the Editor of The Hospital, 28 Southampton Street, Strand, London, W.C. 2, and marked "Bureau of Information."

### INSTITUTION FACTS AND FIGURES.

### Accommodation of Children in London Hospitals.

#### REPORT OF THE MANSION HOUSE COUNCIL.

The Mansion House Council on Health and Nursing, which is amalgamated with the National League for Physical Education and Improvement, has made an inquiry into the adequacy of hospital accommodation and treatment provided for infants and young children in London, and the substance of the information afforded, which refers as far as is practicable to provision in normal times, is summarised in a recently published pamphlet, which may be obtained at the offices of the National League, 4 Tavistock Square, London, W.C. 1.

The inquiry was extended to 44 hospitals and 34 infirmaries, and questions were directed to 106 Infant-Welfare Centres: 41 hospitals, 34 infirmaries, and 49 Welfare Centres replied. As to possible additional beds for infants and young children in hospitals, it was learned that Guy's Hospital is contemplating extension to include 54 cots, and St. Mary's have 31 beds that could be allotted to children if funds were forthcoming. Charing Cross Hospital will reopen a children's ward, now used for soldiers, at the end of the war.

On January 1, 1914, there were 2,389 children in Poor-Law infirmaries in London, and in institutions belonging to the Metropolitan Asylums Board, other than fever and smallpox hospitals, there were 3,043. In infirmaries the proportion of staff to beds is, in most cases, one nurse to eight or ten beds, while at the voluntary hospitals for children the proportion varies between one nurse to two and a-half beds to one nurse to four beds.

Infant-Welfare Centres consider that as regards infirmaries, generally speaking, cases are discharged too soon; the results are unsatisfactory in the case of wasting babies; babies often come out worse than they went in; there is a lack of care, cleanliness, staff, and accommodation, and there is indifferent nursing and medical treatment; infants are sometimes left alone for hours in their cots.

The ideal set up by the Report is that wards should be set aside for infants and young children in existing hospitals, or small local wards set up for minor ailments.

Each Infant-Welfare Centre should have attached to it a residential home or observation ward for wasting babies.

There should be open-air schools for the preventative and curative treatment of consumption.

Minor operation cases ought not to be discharged so quickly as they are now.

Delay in performing operations should be prevented, and the long hours of waiting at the hospital for attendance should be curtailed. Facilities should be available for daily attendance for simple treatment, on the lines of school clinics.

### Special Rations for Invalids.

War bread, it is decided by the Food Ministry's medical advisers, is not generally injurious to invalids. This is a consoling doctrine; but as to buns it would seem to need qualification. The present writer has recently bought in two different shops buns which had a strong flavour of turpentine. Examination led to the conviction that pine-wood sawdust had been mixed with the flour. Additional powers are now conferred upon Food Control Committees which enable them to make grants of extra rations under medical certificate and to issue licences for white flour to patients suffering from a short list only of diseases. In the first case there is no doubt less danger now than in the early days of the war that the fashionable practitioner will readily concede the need of a wealthy patient. No doubt it is also true that those who are used to fare sumptuously every day have in some cases suffered more in health than people accustomed to plain and not too liberal diet. The permission to grant extra rations for a week only, pending decision of an exceptional case by the Ministry's medical advisers, may be useful in an occasional serious emergency.

Extra grants to persons coming from wrecked or torpedoed ships will be grudged by no one. Nor will the extra ration to expectant mothers during the last three months of pregnancy. This is to consist of two meat coupons, or one butter and margarine coupon, weekly, making either a 50 per cent. addition of meat or 100 per cent. to the normal ration of fats. Nursing mothers gain by the provision of the ordinary child's ration from the date of birth to their infants, and in case of the child's death and difficult convalescence of the mother the grant permissible after severe illness or operation may be had as well as that sometimes important item, a priority certificate for milk. At approved Maternity Centres both expectant and nursing mothers may be given, without coupon, meat meals up to 12 oz. weekly. Discharged soldiers generally will be treated as civilians, but medical applications are to have special consideration.

We may add that the real secret of feeding invalids under present conditions is wisdom in catering. The wise housekeeper will provide far more and more attractive nourishment by mere selection. She may, for instance, reject hard meat or a tough fowl, and buy half a calf's head or a whole sheep's head. The meat, tongue, and brains and the resulting broth will furnish a whole series of light, nourishing and attractive dishes. *Verb. sap.*

### SEPTEMBER QUESTION.

### Fuel and Lighting Economies.

1. Describe means by which economies may be effected in the consumption of fuel and lighting in an institution (a) in the wards, ward kitchens, etc., (b) in the kitchens and servants' quarters, (c) in the Nurses' Home, (d) in the administration building.

2. What steps may be taken in the engineer's department to keep up the heating and lighting and hot-water supplies with the least possible expenditure of fuel?

(A minimum payment of 10s. 6d. will be made for each published answer. It is not imperative for both parts of the question to be answered.)

### RULES.

The following rules must be observed:—

1. Contributions must be written on one side of the paper. Brevity and terseness are desirable features. The MSS. must bear the name and address of the sender and be accompanied by coupon to be cut from the back cover (inside page) of the current issue of The Hospital. A pseudonym must be chosen if the name is not to be published.

2. Contributions must be addressed to the Editor of The Hospital, Institutional Worker Supplement, 28 & 29 Southampton Street, Strand, London, W.C. 2, should reach him before the end of the current month, and be marked in the left-hand corner "Facts and Figures."

### QUESTIONS INVITED.

In connection with our Question Box, we offer every month five shillings each for the two best questions which are sent in for consideration. The questions may be on any institutional subject and concern any department, from the secretary's to the porter's, or the matron's to the domestic staff; the one essential is that they must be practical and deal with points that have a definite relation to hospital or institutional administration, upkeep, finance, or management. Points relating to artificers' work, housekeeping, the laundry, out-patients, and other practical matters will be welcome. It is hoped that thus, when difficult or doubtful points arise in the course of their work, institutional workers will be encouraged to put them in the form of a question and send them to the Editor, The Hospital Bureau of Information, 28 & 29 Southampton Street, Strand, W.C. 2, marked "Question Box."

The best questions will be published in due course and our readers will be given the opportunity of answering them. Thus all institutional workers may be able to co-operate with the view of helping the work and smoothing the difficulties of each other. In short, we want the Question Box of the *Institutional Worker* to be freely used by every worker habitually.

### ENQUIRIES AND ANSWERS.

### *EMPLOYMENT AND TRAINING.*

### Six Months' Training in General Hospital.

The London Hospital accepts special pupil probationers on special terms; also St. Bartholomew's, West Smithfield, E.C. The terms at the latter-named institution are £13 13s. per quarter, and laundry anl uniform have to be provided. Write to the matron in each case, who will give you full particulars of training. Obtain "How to Become a Nurse," published by The Scientific Press, Ltd., 28 and 29 Southampton Street, Strand, W.C. 2., price 2s. 10d., post free, which gives a full list of training-schools.—E. M. W.

### Non-Resident Paying Pupil.

A certain number of ladies wishing to obtain some practical experience in sick nursing are received as non-resident paying pupils at Chelsea Infirmary, Cale Street, S.W., for periods of three to six months. A fee of 10s. a week is asked, which includes board and laundry. (It is just possible that the fee is raised slightly now, owing to the increased cost of living.)—Mrs. S.

### General Training in Small Hospital.

You might apply to the West Herts Hospital, Hemel Hempstead, or the Hertford County Hospital. At the first-named institution candidates are accepted for three years' training at the age of twenty. Salary at the rate of £8, £12, £15 a year respectively is given, and laundry and indoor uniform provided. At the last-named institution probationers are received for three years' training; there is no special age or qualification; the salary offered is £10 the first year and £15 and £20 the second and third year respectively. As you ask our advice, we suggest that you wait until you are a little older and then enter a recognised training-school—*e.g.* a hospital of 100 beds or over.—Ada.

### Hospital Administrative Work Training.

Applicants holding a certificate for three years' training in a general hospital containing not less than 200 beds, and who have had subsequent experience as ward sisters, are accepted as pupils for administrative work at Guy's Hospital, London, S.E. The course extends over three months, and comprises housekeeping in hospital and Nurses' Home, the management of kitchens and stores, the ordering and receiving of milk, meat, etc., from tradesmen, the management of servants and their work, the cooking and serving of diets to patients and staff, laundry work, the management of linen stores including stocktaking, book-keeping, and the management of matron's office, including the engagement of probationers and servants. The fee for the course is 18 guineas. The pupil provides her own washing and uniform.—Inquirer.

### *THE SICK AND IN NEED.*

### Open-Air Surgical Home for Crippled Boy.

The St. Vincent's Open-Air Surgical and Industrial Home for Crippled Boys, Eastcote, near Pinner, takes a few free or partly-paid cases. Boys suffering from tuberculous disease of the bones or joints, infantile paralysis, etc., are accepted, and during treatment are educated and given industrial training. The home is under the care of the Sisters of Charity.—Anxious.

### *THE HOUSEKEEPERS' DEPARTMENT.*

### Recipe for Fish Cakes.

Remove all skin and bone from 2 lb. of fish and shred finely. Add to this 1 lb. of mashed potatoes, 1 oz. of margarine or dripping (previously melted), and seasoning to taste, together with some chopped parsley; bind this together with thick white sauce instead of the usual egg binding. Turn out on to a floured board, divide into portions, and brush over with milk and breadcrumbs. Fry in boiling fat to a golden brown.—Kitty.

### Dressing Mackintosh.

An excellent material suitable for dressing mackintoshes can be obtained from the Liverpool Lint Co., Mark Street Mills, Liverpool. It is known as "Impermiette." It will withstand the action of boiling water, carbolic and other antiseptics, and can be readily sterilised.—Mops.

### *MISCELLANEOUS.*

### Laboratory Facilities.

It is certainly desirable that you should equip your hospital with a pathological laboratory. The question of expense may create a difficulty, though not an insurmountable one, if the needs of your institution are properly placed before the public; on the other hand it should be borne in mind that the service of your hospital cannot be efficient without laboratory facilities. As your institution is small it would not be wise to spend a large sum on the equipment for the more elaborate tests which you can get done elsewhere.—L. G. C.

### Static Machine Difficulties.

In this country especially it is essential that the static machine should be enclosed in a glass case. To keep the surrounding air free from moisture probably the best material to use is unslaked lime. If a considerable quantity of this is packed loosely in boxes covered with flannel or cloth it will not require renewal for several months. This, of course, depends upon the condition of the atmosphere. Deposits in the glass case resulting from the ionisation of the air and the formation of nitrous oxide may be removed with a duster moistened with paraffin.—X. Y. Z.

